# Effect of multiple chronic conditions on family doctor contracting in the elderly in China: the moderating role of socioeconomic status

**DOI:** 10.1186/s12889-023-16438-5

**Published:** 2023-08-12

**Authors:** Bo Lv, Ling Zhang, Kai Meng

**Affiliations:** 1 School of Public Health, Capital Medical University, No.10 Xitoutiao, Youanmenwai Street, Fengtai District, Beijing, 100069 China; 2 Beijing Tiantan Hospital, Capital Medical University, No.119 South of the Fourth Ring Road, Fengtai District, Beijing, 100070 China

**Keywords:** The elderly, Family doctor contract services, Socioeconomic status, Influencing factors

## Abstract

**Background:**

China's family doctor contracting service is an important part of deepening the reform of the healthcare systems, aiming to further develop chronic disease management services, enhance the capacity of primary health care services and improve the health of residents. The purpose of this study was to explore the influence of multiple chronic conditions in the elderly on family doctor contracting and whether socioeconomic status played a moderating role.

**Methods:**

A cross-sectional survey was conducted in Beijing, China. A total of 1814 elderly people over 60 years old were included in this study using a whole-group sampling method. The univariate analysis and logistic regression analysis was used to analyze the data.

**Results:**

21.72% of the elderly signed up with family doctors. The multiple chronic conditions was a factor influencing the elderly to sign up with family doctors (OR = 1.44, 95%CI = 1.28–1.61), and the higher the degree of multiple chronic conditions, the stronger willingness to sign up. Socioeconomic status positively moderates the effect of multiple chronic conditions on signing. Also, physical activity intensity (OR = 1.25, 95%CI = 1.03–1.54) and willingness to first visit primary care facilities (OR = 1.38, 95%CI = 1.25–1.54) influenced the elderly to sign up with family doctors.

**Conclusions:**

The elderly with a high degree of multiple chronic conditions, high activity intensity, and a strong willingness to first visit primary care facilities were more likely to sign up with family doctors. The health literacy of the elderly should be further improved, and publicity on the family doctor contracting service policies for the elderly with lower socioeconomic status should be strengthened to guide them to sign up with family doctors. At the same time, the service capacity of primary care facilities should be further improved to meet the health needs of the elderly.

## Background

The global aging problem is prominent, and the aging trend is particularly serious in China, where the seventh population census shows that the number of people aged over 60 reached 264 million accounting for 18.7% of the total population, and the proportion of people aged over 60 is expected to exceed 30% in 2050 [1]. More seriously, the prevalence of chronic diseases among the elderly in China is gradually increasing and is as high as 75.8% [2]. Thus increasing the demand for safe, effective, convenient primary health care services for the elderly, which has a positive role in safeguarding and improving their health [3–5]. Family doctor contracting service (FDCS) is a key initiative to improve the quality of primary health care services, and its development varies from country to country. The United Kingdom required residents to contract with general practitioners when the National Health Care System was established in 1948, and the United States established FDCS systems in the 1960s [6]. Chinese residents tend to go to high-level hospitals, and in order to promote a system of hierarchical diagnosis and treatment and to promote the residents first to visit primary care facilities, China began to emphasize FDCSs in 2009 and is currently in the developmental stage. China's FDCS is based on the team of family doctors in primary care facilities, and based on the principle of voluntary choice by residents, family doctors establish a health record for the residents and provide services such as diagnosis and treatment of common illnesses, health management, preventive vaccination, and referrals by appointment. The FDCSs have a positive effect on residents' health. Yuan et al. showed that family doctors can provide high-quality comprehensive care services for the elderly [7]. The study of Yu et al. and Wang et al. showed that contracted residents strengthened the monitoring of chronic diseases and increased the utilization of primary care facilities [8, 9]. Lai et al. showed that the health-related quality of life of contracted residents was significantly higher than that of uncontracted residents [10]. In 2016, the government proposed to promote key populations such as chronic disease patients and the elderly to contract with family doctors, and the contracting rate should reach 60% by 2020. However, the results of Yang's study showed that the contracting rate of family doctors among the elderly in China was only 28.2% [11], which was still some way from the goal. Therefore, it is necessary to further study the factors that influence the elderly to sign up with family doctors.

There were few articles examining the factors that influence the elderly to sign up with family doctors, but some studies show that the probability of residents signing up with family doctors increases with the increase of age [12, 13]. The number of elderly with chronic diseases is gradually increasing, and if they suffer from two or more chronic diseases at the same time, they are referred to as having multiple chronic conditions (MCCs) [14, 15]. Several survey results have shown that the prevalence of MCCs in the elderly in China has exceeded 50% [16–18]. MCCs increase the risk of disability and reduce the quality of life in the elderly, causing more adverse health outcomes than having a single chronic disease, which has a serious negative impact on physical and mental health [19–21]. Thus the elderly with MCCs have a higher demand for health services and may be more likely to contract with family doctors. Wang's study showed that the elderly want family doctors to provide more chronic disease management services [22]. Yang's study found that the elderly with cardiovascular-metabolic multimorbidity were more willing to contract with family doctors. However, the authors also state in the limitations of the article that this study included only three chronic diseases, and did not examine the effect of the degree of MCCs on contracting [11].

Socioeconomic status is a comprehensive concept, and studies have been conducted to predict socioeconomic status through educational level, income, and occupation [23]. It has been shown that residents of high socioeconomic status were more likely to suffer from MCCs [24, 25]. Fekete's study showed that residents with higher socioeconomic status were more likely to use the services provided by general practitioners [26]. However, it has also been suggested that the prevalence of MCCs may be lower in residents with high socioeconomic status [27, 28]. The elderly with high education and high income may also be more inclined to seek treatment in high-level medical institutions, reduce the use of primary health care services, and are reluctant to sign up with family doctors [29–31]. There are still contradictions in the above research results, and it is worth further investigating whether socioeconomic status has a positive influence on the elderly's contract with family doctors. Being of different socioeconomic status, there may be differences in the contracting of the elderly with MCCs with family doctors. In summary, this study aimed to explore the influence of MCCs on the elderly' contracting with family doctors and to further investigate whether socioeconomic status plays a moderating role.

## Methods

### Study design and questionnaire survey

A cross-sectional survey was conducted from July to August 2021 in Beijing, China. Using a whole-group sampling method, a questionnaire survey was conducted among the elderly aged ≥ 60 years in a community. This community is located in the F district of Beijing, which is a combination of urban and suburban areas, and also has a relatively large elderly population, so it is representative to select the elderly in this community to carry out the survey. According to the sample size calculation formula, $n=\frac{{{u}^{2}}_{a}p\left(1-p\right)}{{\delta }^{2}}$ where *P* = 28.2% (the contracted rate of family doctors among the elderly was 28.2% according to a related study [11]), *δ* = 0.025, *α* = 0.05, *μα* = 1.96, and the required sample size was calculated as 1245 people.

The questionnaire was self-designed according to the literature review and the purpose of the study and consisted of two parts, the first part was demographic information (7 questions), and the second part was exposure factors and disease outcomes (17 questions). The cronbach's α coefficient was 0.927, indicating that the reliability of the questionnaire was good. A questionnaire survey was conducted among the elderly through uniformly trained investigators. A total of 2052 elderly people were investigated in this study. After excluding the questionnaires with logical errors and those with missing values, 1814 valid questionnaires were recovered, meeting the minimum sample size requirement, and the effective recovery rate of the questionnaires was 88.40%.

### Measurements

### Dependent variable

This study was based on the question "Are you currently contracting with family doctors?" to determine the contracting status between the elderly and the family doctors, and answering "yes" meant that the elderly had contracted with family doctors, and answering "no" meant that the elderly had not contracted with family doctors.

### Independent variables

In this study, 12 common chronic diseases (hypertension, diabetes mellitus, dyslipidemia, coronary heart disease, stroke, chronic bronchitis, chronic obstructive pulmonary disease, asthma, chronic hepatitis or cirrhosis, peptic ulcer, benign tumor, and malignant tumor) in the elderly were selected through literature review [18, 32]. According to the number of chronic diseases, there were four levels of suffering from less than (including) one chronic disease, two chronic diseases, three chronic diseases, and greater than (and including) four chronic diseases.

Socioeconomic status is generally a comprehensive indicator including educational level, income, and occupation, and some studies used single or multiple indicators for measurement [23, 33, 34]. However, the classification of occupation is essentially based on educational level and income, and the criteria are more subjective [35]. At the same time, Chinese elderly people over 60 years old have retired, the proportion of re-employment is very low, and the heterogeneity of indicators is not high. So measuring the socioeconomic status of elderly people by occupation has limitations. Income can reflect the guarantee of daily necessities, and educational level means the ability of individuals to obtain resources to meet their needs [36, 37]. Therefore, this study referred to the study of Jiao et al., and combined income (I) and educational level (E) after standardized processing to represent socioeconomic status (SESs) [38].

$${Z}_{i1}=(E-{mean}_{E})/Standard \ {deviation}_{E}$$

$${Z}_{i2}=(I-{mean}_{I})/Standard \ {deviation}_{I}$$

$${SES}_{i}=({Z}_{i1}+{Z}_{i2})/2$$

### Control variables

Based on the literature review, combined with the study purpose and population characteristics of this study, gender, age, marital status, smoking status, drinking status, physical activity intensity, self-rated health, and the willingness to first visit primary care facilities were selected as control variables.

### Data analysis

SPSS 26.0 was used for data analysis in this study. Frequency and percentage were used to describe the demographic characteristics and health-related characteristics of the elderly. The chi-square test to compare differences in basic demographic characteristics and health-related characteristics between contracted and uncontracted older adults. Logistic regression analysis was used to explore the factors influencing the contract between the elderly and family doctors. Model 1 adds control variables, MCCs, and socioeconomic status, and Model 2 adds an interaction term for MCCs and socioeconomic status. Variable assignments are shown in Table 1.

**Table 1:** Variable assignment

Variables	Assignment
Family doctor contracting status	0 = Uncontracted; 1 = Contracted
Multiple chronic conditions	Less than (and including) one chronic disease = 1; Two chronic diseases = 2; Three chronic diseases = 3; Greater than (and including) four chronic diseases = 4
Socioeconomic status	Continuous variable
Gender (male as reference)	
Female	0 = No; 1 = Yes
Age (years) (60–69 years as reference)	
70–79	0 = No; 1 = Yes
80–89	0 = No; 1 = Yes
≥ 90	0 = No; 1 = Yes
Marital status (married as reference)	
Unmarried	0 = No; 1 = Yes
Divorced	0 = No; 1 = Yes
Widowed	0 = No; 1 = Yes
Smoking status (never smoked as reference)	
Used to smoke	0 = No; 1 = Yes
Now smoking	0 = No; 1 = Yes
Drinking status (never drank as reference)	
Used to drink	0 = No; 1 = Yes
Now drinking	0 = No; 1 = Yes
Physical activity intensity	Low degree = 1; Medium degree = 2; High degree = 3
Self-rated health	Very poor = 1; Poor = 2; Relatively poor = 3; General = 4; Relatively good = 5; Good = 6; Very good = 7
Willingness to first visit primary care facilities	Very reluctant = 1; Relatively reluctant = 2; General = 3; Relatively willing = 4; Very willing = 5

## Results

### Basic characteristics of the elderly

Among the surveyed elderly, 391 people (21.72%) were contracted with family doctors and 1423 people (78.28%) were not contracted with family doctors. There were 979 males and 835 females, the largest number of people aged 60–69 was 679 (38.42%), and most of the elderly were married (79.93%). The educational level of the elderly was concentrated in junior high school and high school, with 689 people (37.98%) and 637 people (35.12%) respectively. The monthly income of 815 people (44.93%) and 733 people (40.41%) was 4,000–5,999 yuan and 6,000–7,999 yuan, respectively (Table 2).

**Table 2:** Basic demographic characteristics of the elderly

	Total (*n*=1,814) *n*(%)	Uncontracted (*n*=1,423) *n*(%)	Contracted (*n*=391) *n*(%)	*χ2*	*P*-Value
Gender				9.086	0.028
Male	979 (53.97)	781 (54.88)	198 (50.64)		
Female	835 (46.03)	642 (45.12)	193 (49.36)		
Age (years)				2.225	0.137
60–69	697 (38.42)	539 (37.88)	158 (40.41)		
70–79	567 (31.26)	429 (30.15)	138 (35.29)		
80–89	528 (29.11)	437 (30.71)	91 (23.27)		
≥ 90	22 (1.21)	18 (1.26)	4(1.02)		
Marital status				0.979	0.881
Married	1,450 (79.93)	1,132 (79.55)	318 (81.33)		
Unmarried	11 (0.61)	8 (0.56)	3 (0.77)		
Divorced	32 (1.76)	26 (1.83)	6 (1.53)		
Widowed	321 (17.70)	257 (18.06)	64 (16.37)		
Educational level				4.836	0.436
Illiterate	6 (0.33)	5 (0.35)	1 (0.26)		
Primary school	93 (5.13)	75 (5.27)	18 (4.60)		
Junior high school	689 (37.98)	555 (39.00)	134 (34.27)		
High school	637 (35.12)	493 (34.65)	144 (36.83)		
College and undergraduate	387 (21.33)	293 (20.59)	94 (24.04)		
Postgraduate and above	2 (0.11)	2 (0.14)	0 (0.00)		
Average monthly income (yuan)				7.876	0.096
≤ 1999	2 (0.11)	2 (0.14)	0 (0.00)		
2000–3999	16 (0.88)	12 (0.84)	4 (1.02)		
4000–5999	815 (44.93)	619 (43.50)	196 (50.13)		
6000–7999	733 (40.41)	583 (40.97)	150 (38.36)		
≥ 8000	248 (13.67)	207 (14.55)	41 (10.49)		

### Health status of the elderly

Among all the respondents, 259 (14.28%) of the elderly now smoke, 402 (22.16%) of the elderly now drink, and most of the elderly had high intensity of physical activity (1132 people, 62.40%). About half of the elderly suffer from two or more chronic diseases, with the highest number of elderly suffering from two chronic diseases (454 people, 25.03%). There were 699 people (38.53%) and 257 people (14.17%) who were relatively willing or very willing to first visit primary care facilities, respectively. 713 (39.31%) of the elderly had good self-rated health. There were statistically significant differences in physical activity intensity, MCCs and willingness to first visit primary care facilities between contracted and uncontracted elderly people (Table 3).

**Table 3:** Health status of the elderly

	Total (*n*=1,814) *n*(%)	Uncontracted (*n*=1,423) *n*(%)	Contracted (*n*=391) *n*(%)	*χ*^*2*^	*P*-Value
Smoking status				5.298	0.071
Never smoked	1,326 (73.10)	1,031 (72.45)	295 (75.45)		
Used to smoke	229 (12.62)	175 (12.30)	54 (13.81)		
Now smoking	259 (14.28)	217 (15.25)	42 (10.74)		
Drinking status				0.946	0.623
Never drank	1,308 (72.11)	1,027 (72.17)	281 (71.87)		
Used to drink	104 (5.73)	85 (5.97)	19 (4.86)		
Now drinking	402 (22.16)	311 (21.86)	91 (23.27)		
Physical activity intensity				9.670	0.008
Low degree	130 (7.17)	115 (8.08)	15 (3.84)		
Medium degree	552 (30.43)	438 (30.78)	114 (29.16)		
High degree	1,132 (62.40)	870 (61.14)	262 (67.01)		
Multiple chronic conditions				47.585	< 0.001
≤ 1 chronic disease	935 (51.54)	792 (55.66)	143 (36.57)		
2 chronic diseases	454 (25.03)	335 (23.54)	119 (30.43)		
3 chronic diseases	266 (14.66)	182 (12.79)	84 (21.48)		
≥ 4 chronic diseases	159 (8.77)	114 (8.01)	45 (11.51)		
Self-rated health				5.001	0.660
Very poor	2 (0.11)	2 (0.14)	0 (0.00)		
Poor	17 (0.94)	15 (1.05)	2 (0.51)		
Relatively poor	84 (4.63)	68 (4.78)	16 (4.09)		
General	407 (22.44)	313 (22.00)	94 (24.04)		
Relatively good	713 (39.31)	549 (38.58)	164 (41.94)		
Good	482 (26.57)	389 (27.34)	93 (23.79)		
Very good	109 (6.01)	87 (6.11)	22 (5.63)		
Willingness to first visit primary care facilities				45.189	< 0.001
Very reluctant	97 (5.35)	80 (5.62)	17 (4.35)		
Relatively reluctant	483 (26.63)	421 (29.59)	62 (15.86)		
General	278 (15.33)	226 (15.88)	52 (13.30)		
Relatively willing	699 (38.53)	519 (36.47)	180 (46.04)		
Very willing	257 (14.17)	177 (12.44)	80 (20.46)		

### Analysis of factors influencing the elderly to sign up with family doctors

**Fig. 1:**
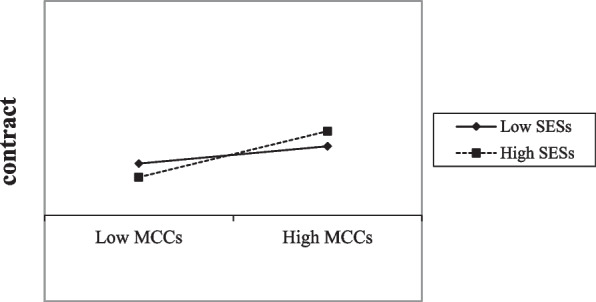
Moderating role of socioeconomic status SESs: socioeconomic status, MCCs: multiple chronic conditions

Model 1 results showed that age at 80–89 years (OR = 0.65, 95% CI = 0.47–0.92), now smoking (OR = 0.56, 95% CI = 0.42–1.00), physical activity intensity (OR = 1.28, 95% CI = 1.05–1.57), willingness to first visit primary care facilities (OR = 1.39, 95% CI = 1.25–1.55) and MCCs (OR = 1.42, 95% CI = 1.27–1.59) influenced the elderly to sign up with family doctors. The higher the degree of MCCs the higher the likelihood of signing up, but socioeconomic status had no significant effect on the elderly to sign up with family doctors. Model 2 added an interaction term for MCCs and socioeconomic status, and the interaction term had a significant positive effect on the elderly signing up with family doctors, suggesting a possible moderating effect of socioeconomic status (Table 4). A further simple slope test showed that the slope of the high socioeconomic status straight line was greater than the slope of the low socioeconomic status straight line, indicating that socioeconomic status has a positive moderating effect in the process of MCCs influencing contracting (Fig. 1).

**Table 4:** Analysis of factors influencing the elderly to sign up with family doctors

Variables	Model 1		Model 2	
	OR	95%CI	OR	95%CI
Gender (male as reference)				
Female	1.05	0.76–1.45	1.05	0.76–1.45
Age (60–69 years as reference)				
70–79	0.90	0.68–1.21	0.90	0.67–1.20
80–89	0.65^*^	0.47–0.92	0.64^*^	0.45–0.89
≥ 90	0.91	0.29–2.90	0.92	0.29–2.92
Marital status (married as reference)				
Unmarried	0.69	0.18–2.68	0.67	0.17–2.61
Divorced	0.54	0.11–2.74	0.54	0.11–2.75
Widowed	0.63	0.16–2.53	0.63	0.16–2.52
Smoking status (never smoked as reference)				
Used to smoke	1.06	0.70–1.62	1.05	0.69–1.60
Now smoking	0.65^*^	0.42–1.00	0.66	0.43–1.01
Drinking status (never drank as reference)				
Used to drink	0.81	0.45–1.48	0.80	0.44–1.47
Now drinking	1.34	0.95–1.89	1.34	0.94–1.89
Physical activity intensity	1.28^*^	1.05–1.57	1.25^*^	1.03–1.54
Self-rated health	1.01	0.89–1.13	1.01	0.90–1.14
Willingness to first visit primary care facilities	1.39^*^	1.25–1.55	1.38^*^	1.25–1.54
Multiple chronic conditions	1.42^*^	1.27–1.59	1.44^*^	1.28–1.61
Socioeconomic status	1.00	0.85–1.18	0.97	0.82–1.14
Multiple chronic conditions × socioeconomic status			1.23^*^	1.07–1.42

^*^ indicates *P* < 0.05

## Discussion

The results of this study indicated that the contracting rate of family doctors among the elderly was 21.72%, which was similar to the results of Yang's study in China [11]. There was still a gap from the goal that the contracting rate of family doctors for key populations should reach 60%, and the FDCSs should be further promoted. The MCCs were an important factor in influencing the elderly to sign up with family doctors, and as the degree of MCCs increases elderly were more likely to sign up with family doctors. Meanwhile, the elderly with MCCs of high socioeconomic status may be more willing to sign up with family doctors.

The results of this study were similar to those of Yang's study, in which MCCs influenced the elderly to contract with family doctors [11]. The MCCs can harm the physical and mental health of the elderly, with the impact increasing as the degree of MCCs increases [39, 40]. FDCSs were helpful to improve the treatment effect of chronic diseases, improve the quality of comprehensive care, and improve the health-related quality of life [7, 9, 41, 42]. Patients with chronic diseases may also be more willing to use health services to meet their health needs [43, 44]. Thus the elderly with MCCs were more likely to sign up with family doctors to improve their health. At the same time, some studies have shown that patients with chronic diseases tend to use the continuous medical services provided by general practitioners [45, 46]. Due to the increasing demand for medical services for the elderly with MCCs, family doctors can provide continuous and effective chronic disease management and basic medical services for them. The elderly with MCCs were more likely to sign up with family doctors, and the willingness to sign contracts increased with the increase in the degree of MCCs.

Socioeconomic status is associated with health inequalities, and the results of this study were similar to Yin’s study, in which socioeconomic status was able to play a moderating role [47]. The elderly with higher socioeconomic status may have more resources and opportunities to obtain health information and can learn about chronic disease management and regular follow-up services provided by family doctors through various channels [48]. When these elderly people suffer from chronic diseases, they may be more likely to accept and use FDCSs. Chronic diseases affect the health level of the elderly, and residents with higher socioeconomic status may be more proactive in utilizing healthcare services to improve their health [49]. Therefore, high socioeconomic status may promotes the elderly with chronic diseases to sign up with family doctors. Compared to the elderly of high socioeconomic status, the elderly of low socioeconomic status may have access to fewer health information resources, have slightly lower health literacy, and may have insufficient awareness of FDCSs [50]. At the same time, the elderly with lower socioeconomic status may pay relatively less attention to their health status, have a low percentage of visits to medical institutions, and may tend to alleviate adverse health effects of chronic diseases through self-medication [51, 52]. Thus, low socioeconomic status may discourage the elderly with chronic diseases to sign up with family doctors.

The elderly who were willing to receive the first treatment in the primary care facilities may have a better understanding of the hierarchical treatment system and FDCS policies [53]. They may have more trust in the doctors in the primary care facilities and have higher satisfaction, and be willing to sign up with family doctors [54, 55]. Although family doctors in primary care facilities may not be able to completely solve the health problems of patients, two-way referral channels are currently smooth, and family doctors can transfer patients to high-level hospitals for convenient medical treatment [56]. Some studies showed that residents with high activity intensity generally have better physical fitness and health, and also make more use of public health services, and fewer visits to hospitals [57–59]. Similar to the findings of Nie et al., and Li et al., physical activity was the factor that influenced the elderly' contracts with family doctors [60, 61]. The elderly who are physically active may be more health-conscious and want to improve their health by signing up with family doctors.

### Limitations

First, this study was a cross-sectional study and the relationships of the variables in the results were more likely to be interpreted as correlations, and causal inferences are somewhat limited. Second, the number of chronic diseases in the elderly was calculated by self-reporting whether they had 12 common chronic diseases, and recall bias may exist. Finally, our study was conducted only in Beijing, China, where economic conditions are better, and FDCSs are developing rapidly, so the generalization of the findings of this study is limited, and sampling can be conducted in wider areas in a follow-up study.

## Conclusion

This study provides further evidence that MCCs were a factor that influenced the elderly to sign up with family doctors. Also, as the degree of MCCs increased, the willingness of the elderly to sign up increased. This study found that socioeconomic status was not a factor that directly influenced the elderly to sign up with family doctors, but socioeconomic status positively moderates the influence of MCCs on contracting. Meanwhile, this study found that the elderly with high physical activity intensity and willingness to first visit primary care facilities were more likely to sign up with family doctors. On the one hand, it suggests that the elderly who may be more concerned about their physical health may make more use of primary health care services. On the other hand, it suggests that maybe the capacity of primary care facilities influences the elderly to sign up. It is recommended to strengthen the promotion of the FDCS policies for the elderly with lower socioeconomic status, improve the construction of hierarchical diagnosis and treatment systems, enhance the service capacity of primary care facilities to meet the needs of the elderly and promote the elderly to contract with family doctors.

## Data Availability

The datasets generated and analyzed during the current study are available from the corresponding author on reasonable request. E-mail: mengkai@ccmu.edu.cn.

## Abbreviations

FDCSs: Family doctor contract services
MCCs: Multiple chronic conditions
SESs: Socioeconomic status
